# Impact of the COVID-19 Pandemic on Manual Therapy Service Utilization within the Australian Private Healthcare Setting

**DOI:** 10.3390/healthcare8040558

**Published:** 2020-12-13

**Authors:** Reidar P. Lystad, Benjamin T. Brown, Michael S. Swain, Roger M. Engel

**Affiliations:** 1 Australian Institute of Health Innovation, Macquarie University, Sydney 2109, Australia; 2 Department of Chiropractic, Macquarie University, Sydney 2109, Australia; benjamin.brown@mq.edu.au (B.T.B.); michael.swain@mq.edu.au (M.S.S.); roger.engel@mq.edu.au (R.M.E.)

**Keywords:** COVID-19, health services, cost, manual therapy, chiropractic, osteopathy, physiotherapy

## Abstract

The COVID-19 pandemic has impacted a wide range of health services. This study aimed to quantify the impact of the COVID-19 pandemic on manual therapy service utilization within the Australian private healthcare setting during the first half of 2020. Quarterly data regarding the number and total cost of services provided were extracted for each manual therapy profession (i.e., chiropractic, osteopathy, and physiotherapy) for the period January 2015 to June 2020 from the Australian Prudential Regulation Authority. Time series forecasting methods were used to estimate absolute and relative differences between the forecasted and observed values of service utilization. An estimated 1.3 million (13.2%) fewer manual therapy services, with a total cost of AUD 84 million, were provided within the Australian private healthcare setting during the first half of 2020. Reduction in service utilization was considerably larger in the second quarter (21.7%) than in the first quarter (5.7%), and was larger in physiotherapy (20.6%) and osteopathy (12.7%) than in chiropractic (5.2%). The impact varied across states and territories, with the largest reductions in service utilization observed in New South Wales (17.5%), Australian Capital Territory (16.3%), and Victoria (16.2%). The COVID-19 pandemic has had a profound impact on manual therapy service utilization in Australia. The magnitude of the decline in service utilization varied considerably across professions and locations. The long-term consequences of this decline in manual therapy utilization remain to be determined.

## 1. Introduction

The novel coronavirus disease caused by severe acute respiratory syndrome coronavirus 2 (SARS-CoV-2) that emerged in Wuhan, China, in December 2019 (COVID-19) was declared a global pandemic by the World Health Organization on 11 March 2020 [1]. Major human events and natural calamities such as global pandemics have the potential to affect human behavior and access to resources, including healthcare seeking behavior and service utilization. There is emerging evidence that the COVID-19 pandemic has impacted a wide range of health services, including stroke emergency services [2,3,4], medical imaging services [5,6], and hospice care [7]. However, the impact of the COVID-19 pandemic on manual therapy service utilization is unknown.

In Australia, chiropractors, osteopaths, and physiotherapists are registered healthcare practitioners trained to diagnose, treat, and manage patients with musculoskeletal conditions. As of the first quarter of 2020, there were 5383 chiropractors, 2627 osteopaths, and 33,299 physiotherapists with general registration to practice in Australia [8,9,10]. The manual therapy services provided by these professions are predominately paid for by non-government sources (i.e., private health insurers and individuals). Studies have documented increased utilization of manual therapy services over time in Australia, albeit with diverging trends across professions [11,12]. It has been estimated that approximately 21.6 million manual therapy services with a total cost of AUD 1.4 billion were provided within the Australian private healthcare setting annually in the period between 2013 and 2017, which represented a significant increase from the preceding five-year period [12]. It remains to be determined how these trends and figures have been impacted by the COVID-19 pandemic.

This study aimed to quantify the impact of the COVID-19 pandemic on manual therapy service utilization within the private healthcare setting in Australia. The specific objectives were to quantify the absolute and relative difference between forecasted and observed number and total cost of services during the first half of 2020 for each manual therapy profession.

## 2. Materials and Methods

### 2.1. Data Sources

Statistics on private health insurance industry activity in Australia are available from the Australian Prudential Regulation Authority (APRA) [13]. For the present study, we extracted quarterly data on the number and total cost of services for each manual therapy profession (i.e., chiropractic, osteopathy, and physiotherapy) from the first quarter (Q1) of 2015 to the second quarter (Q2) of 2020. We also extracted quarterly data on the estimated number of persons covered under private health insurance general treatment cover. A pyramid plot of the number of persons insured by sex and age group in 2020 Q2 is provided in Section A of the Supplementary Materials (Figure S1). Statistics on registered healthcare practitioners are available from the Australian Health Practitioner Regulation Agency (AHPRA) and each profession’s registration board [8,9,10,14]. For this study, we extracted quarterly data on the number of registered providers from each manual therapy profession from 2015 Q1 to 2020 Q2. We did not include registrants listed as limited or non-practicing. We then estimated the number of providers working in the private sector for each profession as follows: For chiropractic and osteopathy, 100% of registrants were taken to be working in the private sector because neither of these professions contribute to service provision in the public sector. For physiotherapy, 63.5% of registrants were taken to be working in the private sector, as estimated by a National Health Workforce Report [15].

### 2.2. Operational Definitions

There are three types of private health insurance coverage in Australia: hospital treatment only, general treatment only, and combined hospital and general treatment. General treatment cover includes most services for preventing or managing injuries, diseases, and conditions that are provided outside of the hospital setting. However, it excludes services for chronic disease management that are covered by Medicare. A service was defined as one visit to a healthcare provider.

### 2.3. Data Management and Analysis

All dollar values were adjusted for inflation using the Reserve Bank of Australia’s online inflation calculator and reported in second quarter of 2020 Australian dollars [16]. The main outcome variables were number of services per quarter and total cost of services per quarter. The number of providers was used as the denominator to calculate the following secondary outcome variables: number of services per provider per quarter and total cost per provider per quarter. The number of individuals with general treatment cover was used as the denominator to calculate the following secondary outcome variables: number of services per 100,000 insured population per quarter and total cost of services per 100,000 insured population per quarter.

Time series forecasting involved fitting seasonal autoregressive integrated moving average (ARIMA) models of service utilization data from 2015 to 2019 using the methods described by Hyndman and Athanasopoulos [17]. The seasonal ARIMA models provided estimates that account for seasonality and trends over time. Point forecast estimates with 95% prediction intervals for 2020 Q1 and Q2 were calculated from the seasonal ARIMA models and compared against observed values. The resulting mean errors and mean percentage errors were used as measures of absolute and relative impact. All statistical analyses were conducted using R, version 3.5.1 (R Foundation for Statistical Computing, Vienna, Austria) using the forecast and hts packages.

## 3. Results

For the three manual therapy professions combined, an estimated 1,322,370 (13.2%) fewer services were provided during the first half of 2020. The estimated reduction in total cost of services provided amounted to AUD 83,972,816 (11.5%). The combined estimated relative reduction in quarterly number and total cost of services provided was greater in Q2 (21.7% and 16.6%, respectively) than in Q1 (5.7% and 6.8%, respectively).

**Figure 1:**
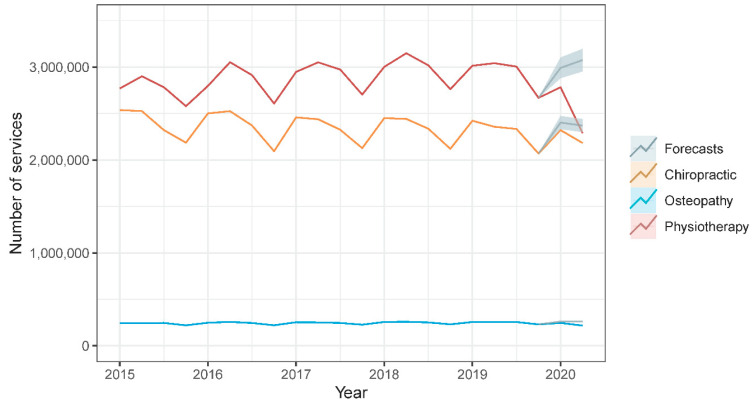
Time series plot of observed values and point forecast estimates with 95% prediction intervals of the quarterly number of services provided by each manual therapy profession from 2015 to 2020 Q2.

**Figure 2:**
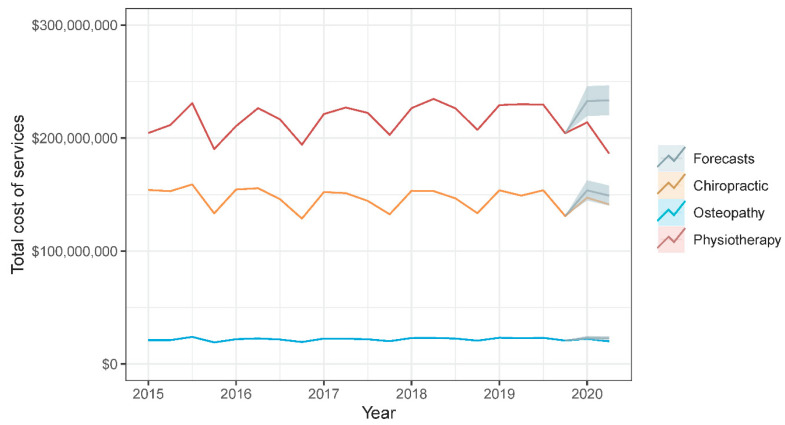
Time series plot of observed values and point forecast estimates with 95% prediction intervals of the quarterly total cost of services provided by each manual therapy profession from 2015 to 2020 Q2.

During the first half of 2020, the estimated relative reduction in number of services provided was considerably larger in physiotherapy (20.6%) and osteopathy (12.7%) than in chiropractic (5.2%). Figure 1 shows a time series plot of observed values and point forecast estimates with 95% prediction intervals of the quarterly number of services provided by each manual therapy profession from 2015 to 2020 Q2. Similarly, the estimated relative reduction in the quarterly total cost of services provided was considerably larger in physiotherapy (17.0%) and osteopathy (13.1%) than in chiropractic (4.7%). Figure 2 shows a time series plot of observed values and point forecast estimates with 95% prediction intervals of the quarterly total cost of services provided by each manual therapy profession from 2015 to 2020 Q2. Table 1 provides an overview of observed values, point forecast estimates, mean absolute error, and mean absolute percentage error of the quarterly number and total cost of services provided by each manual therapy profession during 2020 Q1 and Q2.

**Figure 3:**
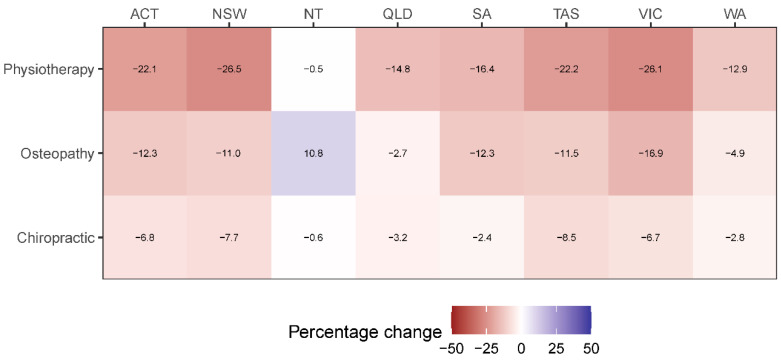
Heatmap of the estimated mean percentage error in number of services provided by each manual therapy profession during the first half of 2020 across Australian states and territories.

**Figure 4:**
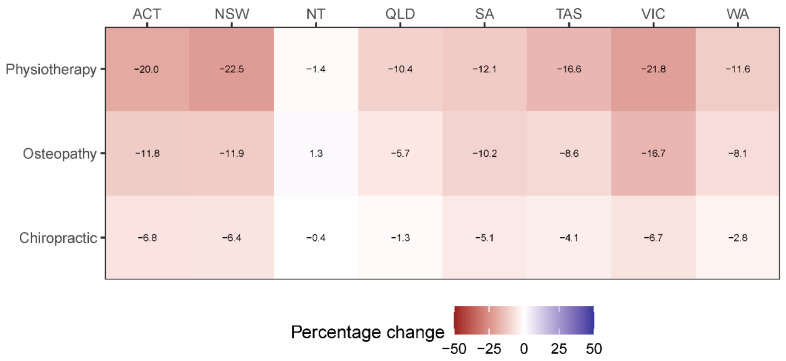
Heatmap of the estimated mean percentage error in total cost of services provided by each manual therapy profession during the first half of 2020 across Australian states and territories.

The estimated relative reduction in number and cost of services provided during the first half of 2020 varied by Australian state and territory. The estimated relative reduction in number of services provided was largest in New South Wales (17.5%), followed by Australian Capital Territory (16.3%), Victoria (16.2%), Tasmania (15.0%), South Australia (10.3%), Queensland (8.7%), Western Australia (7.9%), and Northern Territory (0.3%). Similarly, the estimated relative reduction in total cost of services provided was largest in New South Wales (15.6%) and Australian Capital Territory (15.6%), followed by Victoria (15.0%), Tasmania (11.2%), South Australia (9.3%), Western Australia (7.9%), Queensland (6.4%), and Northern Territory (0.9%). Figure 3 and Figure 4 are heatmaps depicting the estimated mean percentage error in number and total cost of services, respectively, provided by the three manual therapy professions during the first half of 2020 across Australian states and territories.

Because the number of individuals with general treatment cover and the number of manual therapy providers varied from quarter to quarter, supplementary analyses were conducted using secondary outcome variables and are presented in the Supplementary Materials (Sections B and C, respectively). These supplementary analyses generated similar estimates of the percentage change in manual therapy service utilization.

## 4. Discussion

This is the first study to estimate the impact of the COVID-19 pandemic on manual therapy service utilization. During the first half of 2020, the COVID-19 pandemic coincided with approximately 1.3 million fewer chiropractic, osteopathy, and physiotherapy services provided within the Australian private healthcare system. The associated loss of revenue was estimated to be AUD 84 million. Physiotherapy incurred the largest relative reduction in service provision and revenue, while chiropractic was found to be the least impacted of the three manual therapy professions. Geographically, the largest relative reductions in manual therapy service utilization were observed in the south-eastern corner of mainland Australia (i.e., New South Wales, Australian Capital Territory, and Victoria).

Our findings show that the COVID-19 pandemic has had a profound impact on manual therapy service utilization in Australia. It is not unexpected to observe disruption of health services during a global pandemic. While much of the initial disruption of health services has been characterized by the surge in demand for front-line care of COVID-19 patients, there has also been a concomitant reduction in or discontinuation of prevention and rehabilitation services for noncommunicable diseases. For instance, the World Health Organization reported that more than half of the countries surveyed had partially or completely disrupted services for the treatment or clinical management of hypertension, diabetes and diabetes-related complications, cancer, and cardiovascular emergencies [18]. A similar situation exists in Australia, where there have been marked reductions in a wide range of health services, including breast and prostate cancer screenings [19,20], pediatric orthopedic hospital services [21], trauma care in Emergency Departments [22,23], and initiation of cardiopulmonary resuscitation by Emergency Medical Services in public areas [24]. It is important to note that our findings encompass only the initial disruption caused by the COVID-19 pandemic. It remains to be determined whether the decline in manual therapy service utilization persists beyond the first half of 2020, whether manual therapy service utilization returns to pre-pandemic levels after the virus has been eliminated, and whether economic factors such as sustained or transient changes in disposable income, uptake of private health insurance, and cost of services have any lasting effects on healthcare-seeking behavior and manual therapy service utilization. Future studies are encouraged to explore these unresolved questions.

The magnitude of the decline in manual therapy service utilization during the COVID-19 pandemic was not uniform across the three professions, with physiotherapy and chiropractic experiencing the largest and smallest relative reductions, respectively. The exact reason for this difference is unclear, but it may be related to differences in revenue streams (e.g., proportion derived from private health insurance) and patient characteristics (e.g., level of disposable income) across the three professions [12]. For instance, as the COVID-19 pandemic has unfolded and loss of income became more prevalent in the population, perhaps Australians have forfeited their private health insurance with general treatment cover as a cost-saving measure. While this explanation may sound reasonable given the circumstances, it does not explain why there was a larger decline in physiotherapy service utilization relative to chiropractic and osteopathy. Nor does it explain the fact that the results from our supplementary analyses of service utilization per 100,000 insured persons were very similar to our main findings, which suggests that any changes in the number of insured persons are unlikely to explain the observed differences. An alternative explanation may be differences in case-mix across the three manual therapy professions. Although this explanation is not supported by the scope of practice of the three professions, which all state that they diagnose and treat musculoskeletal conditions, industry reports indicate that physiotherapy provides more specialized services (e.g., neurological rehabilitation, geriatric services, and sports injury prevention and rehabilitation) than osteopathy and chiropractic [25,26]. Thus, it is conceivable that public health orders and social restrictions related to the COVID-19 pandemic (e.g., stay-at-home directives, limited access to aged care facilities, and shutdown of community sport activities) may have resulted in a marked reduction in utilization of specialist physiotherapy services. Although this may explain the larger relative reduction in physiotherapy service utilization (20.6%), it does not adequately explain the differences in relative reduction in services between osteopathy (12.7%) and chiropractic (5.2%). Further research is needed to examine the factors influencing healthcare-seeking behavior and manual therapy service utilization during the COVID-19 pandemic and to elucidate the underlying reasons for the observed differences across the three manual therapy professions.

There are strengths and limitations of the present study. This research builds on our previous reports of manual therapy service utilization within the Australian private healthcare setting [11,12]. We added an inflation adjustment for all dollar values to remove the effect of inflation from our analyses. More importantly, we applied sophisticated analytical techniques to produce forecasts that account for both seasonal variation and long-term trends across professions and geographical regions (i.e., states and territories). In an attempt to account for changes in the number of providers and people with private health insurance, we provided supplementary analyses using secondary outcome variables (i.e., number and total cost of services provided per provider and per 100,000 persons with private health insurance). These supplementary analyses produced very similar estimates of the impact of the COVID-19 pandemic on manual therapy service utilization. Lastly, because our study was limited to private health insurance data, the estimates presented herein can only be generalized to services provided under private health insurance general treatment cover.

## 5. Conclusions

The COVID-19 pandemic has had a profound impact on manual therapy service utilization in Australia. The magnitude of the decline in service utilization varied considerably across professions and locations. The long-term consequences of this decline in manual therapy utilization remains to be determined.

## Figures and Tables

**Table 1:** Observed values, forecast point estimates, mean errors, and mean percentage errors of the quarterly number and total cost of services provided by each manual therapy profession in Australia during 2020 Q1 and Q2.

	Chiropractic	Osteopathy	Physiotherapy
**2020 Q1**			
*Number of services*			
Observed	2,321,874	246,011	2,783,058
Forecast point estimate (95% prediction interval)	2,402,991 (2,332,444 to 2,473,537)	260,956 (253,739 to 268,173)	2,992,101 (2,880,914 to 3,103,288)
Mean error	−81,117	−14,945	−209,043
Mean percentage error	−3.5%	−6.1%	−7.5%
*Total cost*			
Observed	AUD 147,289,851	AUD 22,268,311	AUD 213,939,030
Forecast point estimate (95% prediction interval)	AUD 153,756,465 (AUD 144,918,266 to 162,594,663)	AUD 23,253,647 (AUD 21,596,951 to 24,910,343)	AUD 232,693,899 (AUD 219,505,714 to 245,882,084)
Mean error	−AUD 6,466,613	−AUD 985,336	−AUD 18,754,869
Mean percentage error	−4.4%	−4.4%	−8.8%
**2020 Q2**			
*Number of services*			
Observed	2,183,321	217,595	2,289,773
Forecast point estimate (95% prediction interval)	2,370,623 (2,300,077 to 2,441,170)	261,755 (254,538 to 268,972)	3,075,576 (2,954,135 to 3,197,016)
Mean error	−187,302	−44,160	−785,803
Mean percentage error	−8.6%	−20.3%	−34.3%
*Total cost*			
Observed	AUD 141,323,394	AUD 20,076,981	AUD 186,367,287
Forecast point estimate (95% prediction interval)	AUD 149,092,929 (AUD 140,254,731 to 157,931,128)	AUD 22,939,482 (AUD 21,282,786 to 24,596,178)	AUD 233,501,248 AUD 220,313,063 to 246,689,433)
Mean error	−AUD 7,769,535	−AUD 2,862,502	−AUD 47,133,961
Mean percentage error	−5.5%	−14.3%	−25.3%

## Supplementary Materials

The following are available online at https://www.mdpi.com/2227-9032/8/4/558/s1, Section A: Figure S1: Pyramid plot depicting the number of persons insured under general treatment cover by sex and age group in the second quarter of 2020; Section B: Figure S2: Time series plot of observed values and point forecast estimates with 95% prediction intervals of the quarterly number of services provided per 100,000 insured persons by each manual therapy profession from 2015 to 2020 Q2; Figure S3: Time series plot of observed values and point forecast estimates with 95% prediction intervals of the quarterly total cost of services provided per 100,000 insured persons by each manual therapy profession from 2015 to 2020 Q2; Table S1: Observed values, forecast point estimates with 95% prediction intervals, mean errors, and mean percentage errors of the quarterly number and total cost of services provided per 100,000 insured persons by each manual therapy profession in Australia during 2020 Q1 and Q2; Figure S4: Heatmap of the estimated mean percentage error in number of services provided per 100,000 insured persons by each manual therapy profession during the first half of 2020 across Australian states and territories; Figure S5: Heatmap of the estimated mean percentage error in total cost of services provided per 100,000 insured persons by each manual therapy profession during the first half of 2020 across Australian states and territories; Section C: Figure S6: Time series plot of observed values and point forecast estimates with 95% prediction intervals of the quarterly number of services provided per provider by each manual therapy profession from 2015 to 2020 Q2; Figure S7: Time series plot of observed values and point forecast estimates with 95% prediction intervals of the quarterly total cost of services provided per provider by each manual therapy profession from 2015 to 2020 Q2; Table S2: Observed values, forecast point estimates with 95% prediction intervals, mean errors, and mean percentage errors of the quarterly number and total cost of services provided per provider by each manual therapy profession in Australia during 2020 Q1 and Q2; Figure S8: Heatmap of the estimated mean percentage error in number of services provided per provider by each manual therapy profession during the first half of 2020 across Australian states and territories; Figure S9: Heatmap of the estimated mean percentage error in total cost of services provided per provider by each manual therapy profession during the first half of 2020 across Australian states and territories.
