# Supplementary material for: Impact of the COVID-19 Pandemic on Manual Therapy Service Utilization within the Australian Private Healthcare Setting
Source: Healthcare (Basel). 2020 Dec 13;8(4):558. doi: 10.3390/healthcare8040558 (PMC7764415; doi:10.3390/healthcare8040558)

Supplementary Materials

Table of Contents

Section A: Demographics of population with private health insurance 2

Supplementary Figure S1: Pyramid plot depicting the number of persons insured under general treatment cover by sex and age group in the second quarter of 2020 2

Section B: Supplementary analyses per 100,000 insured persons 3

Supplementary Figure S2: Time series plot of observed values and point forecast estimates with 95% prediction intervals of the quarterly number of services provided per 100,000 insured persons by each manual therapy profession from 2015 to 2020 Q2 3

Supplementary Figure S3: Time series plot of observed values and point forecast estimates with 95% prediction intervals of the quarterly total cost of services provided per 100,000 insured persons by each manual therapy profession from 2015 to 2020 Q2 4

Supplementary Table S1: Observed values, forecast point estimates with 95% prediction intervals, mean errors, and mean percentage errors of the quarterly number and total cost of services provided per 100,000 insured persons by each manual therapy profession in Australia during 2020 Q1 and Q2.. 5

Supplementary Figure S4: Heatmap of the estimated mean percentage error in number of services provided per 100,000 insured persons by each manual therapy profession during the first half of 2020 across Australian States and Territories 6

Supplementary Figure S5: Heatmap of the estimated mean percentage error in total cost of services provided per 100,000 insured persons by each manual therapy profession during the first half of 2020 across Australian States and Territories 7

Section C: Supplementary analyses per provider 8

Supplementary Figure S6: Time series plot of observed values and point forecast estimates with 95% prediction intervals of the quarterly number of services provided per provider by each manual therapy profession from 2015 to 2020 Q2 8

Supplementary Figure S7: Time series plot of observed values and point forecast estimates with 95% prediction intervals of the quarterly total cost of services provided per provider by each manual therapy profession from 2015 to 2020 Q2 9

Supplementary Table S2: Observed values, forecast point estimates with 95% prediction intervals, mean errors, and mean percentage errors of the quarterly number and total cost of services provided per provider by each manual therapy profession in Australia during 2020 Q1 and Q2 10

Supplementary Figure S8: Heatmap of the estimated mean percentage error in number of services provided per provider by each manual therapy profession during the first half of 2020 across Australian States and Territories 11

Supplementary Figure S9: Heatmap of the estimated mean percentage error in total cost of services provided per provider by each manual therapy profession during the first half of 2020 across Australian States and Territories 12

Section A: Demographics of population with private health insurance

Supplementary Figure S1: Pyramid plot depicting the number of persons insured under general treatment cover by sex and age group in the second quarter of 2020

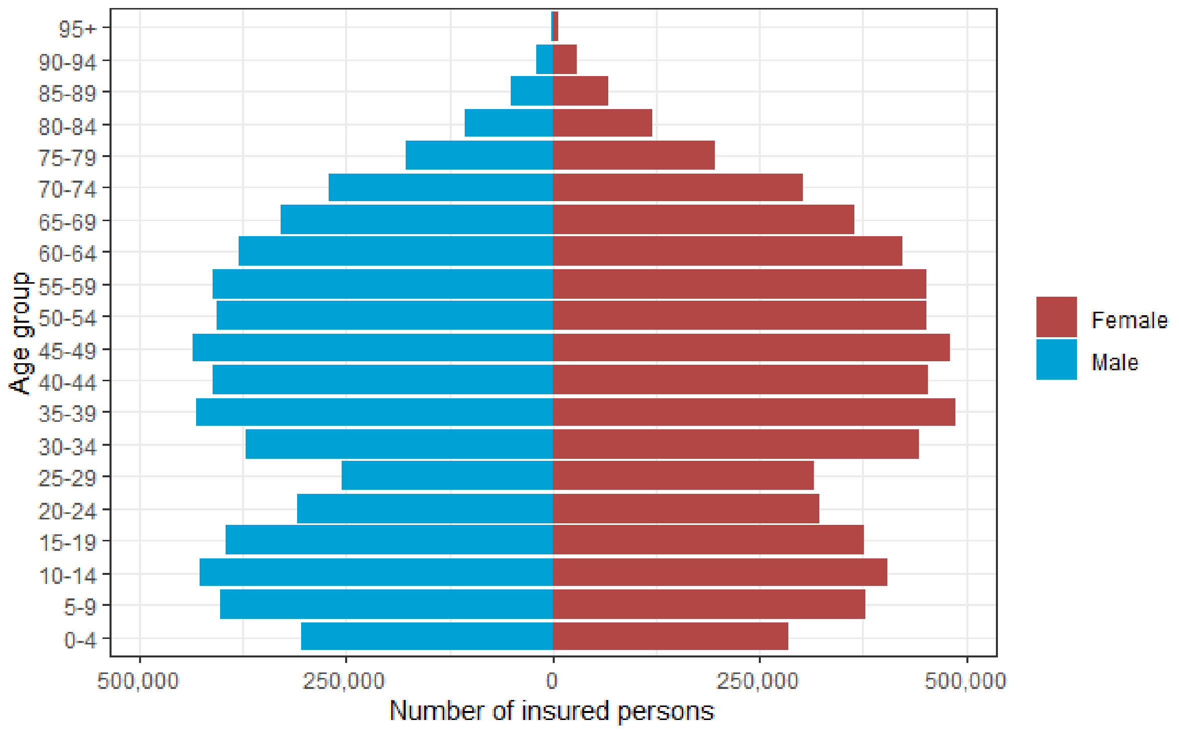

Section B: Supplementary analyses per 100,000 insured persons

Supplementary Figure S2: Time series plot of observed values and point forecast estimates with 95% prediction intervals of the quarterly number of services provided per 100,000 insured persons by each manual therapy profession from 2015 to 2020 Q2

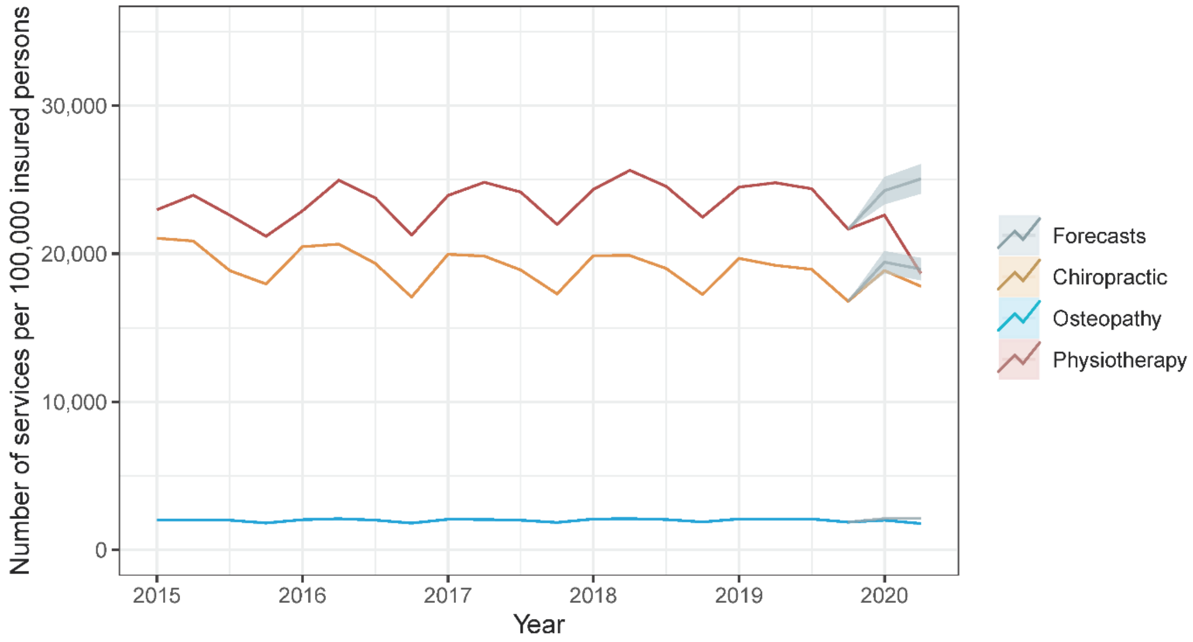

Supplementary Figure S3: Time series plot of observed values and point forecast estimates with 95% prediction intervals of the quarterly total cost of services provided per 100,000 insured persons by each manual therapy profession from 2015 to 2020 Q2

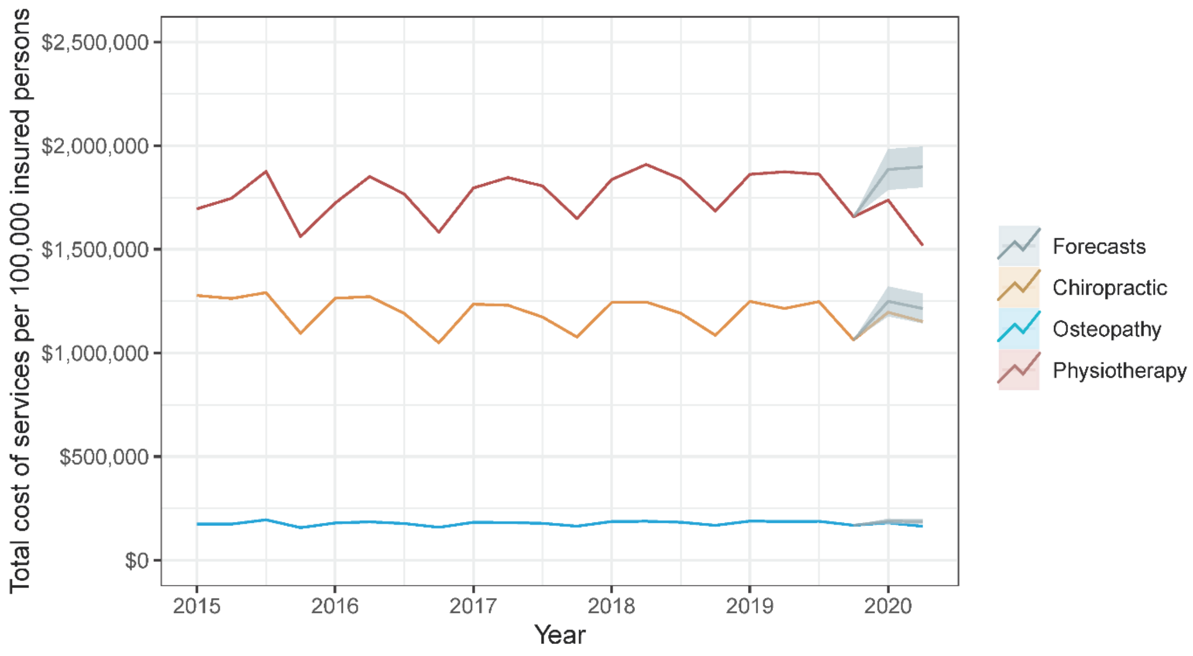

Supplementary Table S1: Observed values, forecast point estimates with 95% prediction intervals, mean errors, and mean percentage errors of the quarterly number and total cost of services provided per 100,000 insured persons by each manual therapy profession in Australia during 2020 Q1 and Q2

	Chiropractic	Osteopathy	Physiotherapy
**2020 Q1**			
Number of services			
Observed	18,860	1,998	22,606
Forecast point estimate	19,429	2,109	24,259
95% prediction interval	18,675 to 20,184	2,062 to 2,157	23,340 to 25,179
Mean error	-569	-111	-1,653
Mean percentage error	-3.0%	-5.6%	-7.3%
Total cost			
Observed	$1,196,391	$180,879	$1,737,762
Forecast point estimate	$1,249,241	$188,931	$1,885,787
95% prediction interval	$1,177,381 to $1,321,101	$176,536 to $201,326	$1,787,601 to $1,983,973
Mean error	-$52,850	-$8,053	-$148,025
Mean percentage error	-4.4%	-4.5%	-8.5%
**2020 Q2**			
Number of services			
Observed	17,796	1,774	18,664
Forecast point estimate	18,960	2,126	25,054
95% prediction interval	18,205 to 19,714	2,078 to 2,173	24,055 to 26,052
Mean error	-1,164	-352	-6,390
Mean percentage error	-6.5%	-19.8%	-34.2%
Total cost			
Observed	$1,151,911	$163,645	$1,519,059
Forecast point estimate	$1,215,035	$186,946	$1,898,031
95% prediction interval	$1,143,175 to $1,286,896	$174,551 to $199,341	$1,799,845 to $1,996,217
Mean error	-$63,124	-$23,300	-$378,972
Mean percentage error	-5.5%	-14.2%	-24.9%

Supplementary Figure S4: Heatmap of the estimated mean percentage error in number of services provided per 100,000 insured persons by each manual therapy profession during the first half of 2020 across Australian States and Territories

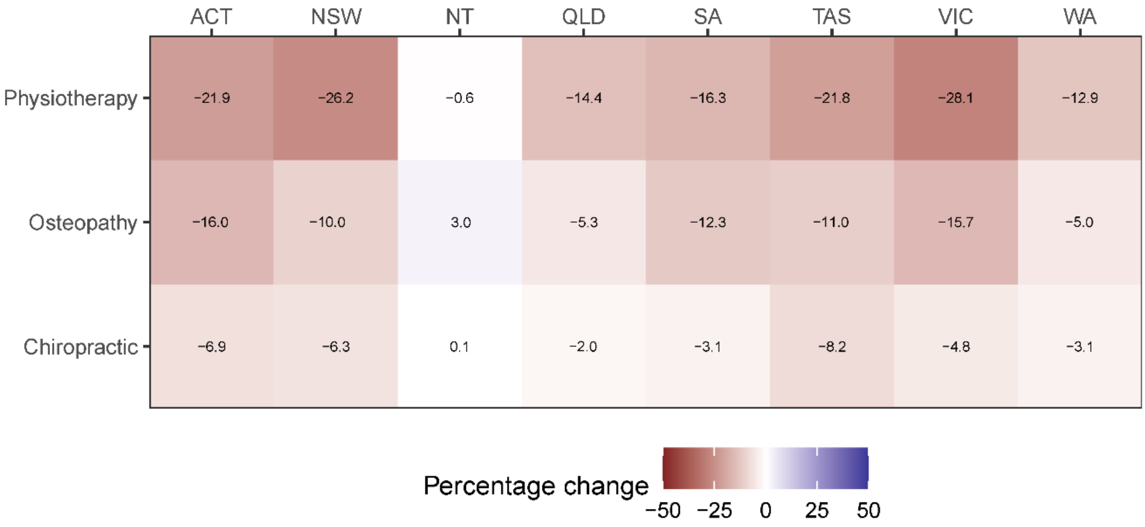

Supplementary Figure S5: Heatmap of the estimated mean percentage error in total cost of services provided per 100,000 insured persons by each manual therapy profession during the first half of 2020 across Australian States and Territories

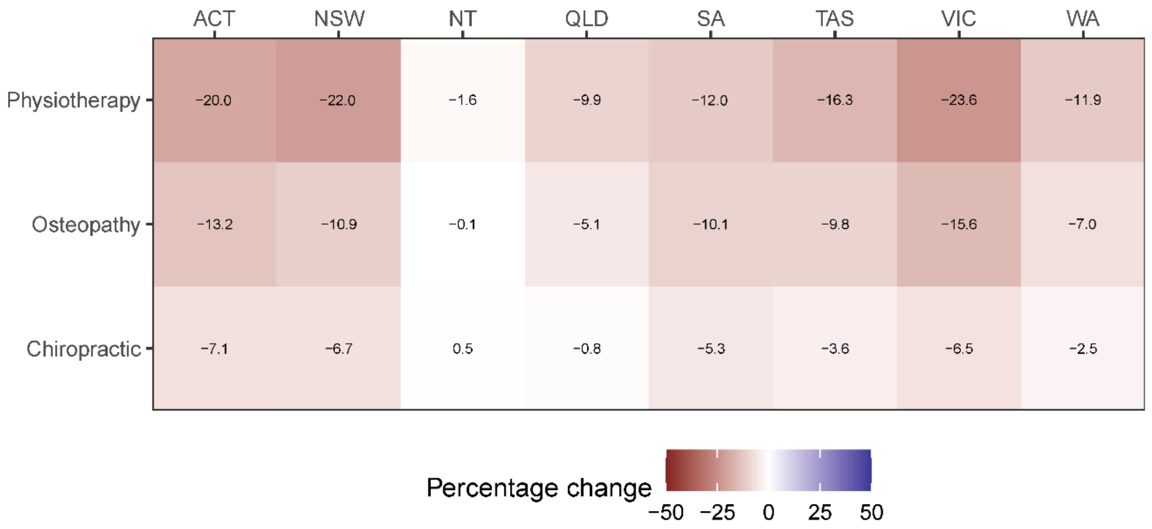

Section C: Supplementary analyses per provider

Supplementary Figure S6: Time series plot of observed values and point forecast estimates with 95% prediction intervals of the quarterly number of services provided per provider by each manual therapy profession from 2015 to 2020 Q2

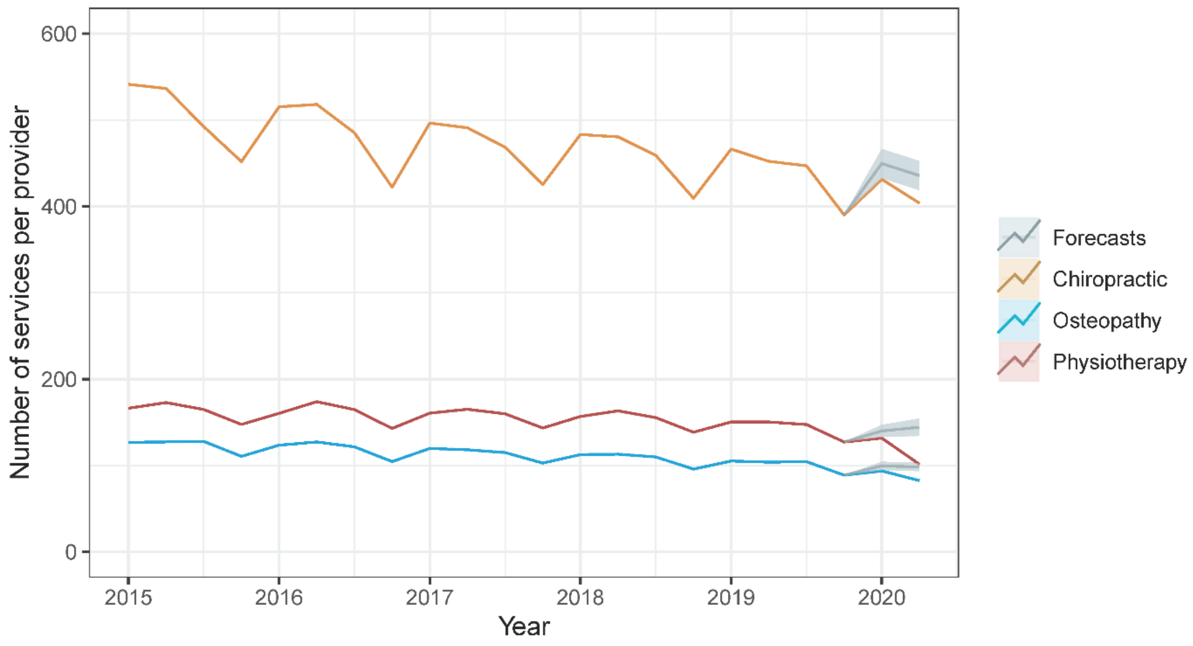

Supplementary Figure S7: Time series plot of observed values and point forecast estimates with 95% prediction intervals of the quarterly total cost of services provided per provider by each manual therapy profession from 2015 to 2020 Q2

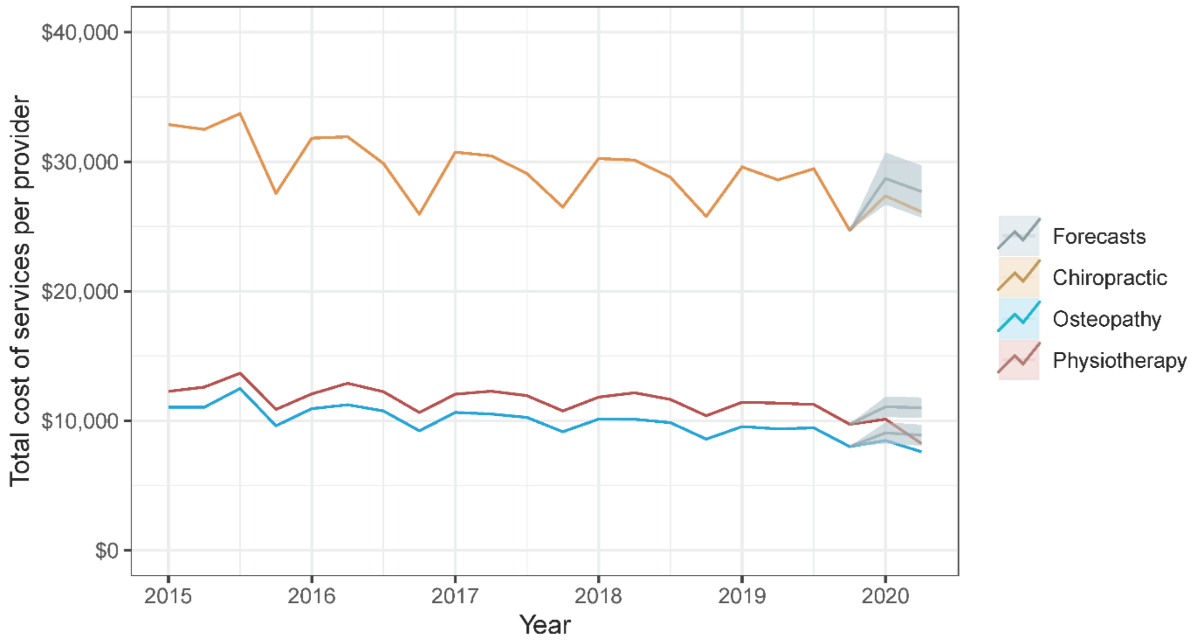

Supplementary Table S2: Observed values, forecast point estimates with 95% prediction intervals, mean errors, and mean percentage errors of the quarterly number and total cost of services provided per provider by each manual therapy profession in Australia during 2020 Q1 and Q2

	**Chiropractic**	**Osteopathy**	**Physiotherapy**
**2020 Q1**			
Number of services			
Observed	431.3	93.6	131.6
Forecast point estimate	449.6	99.5	139.9
95% prediction interval	432.7 to 466.5	94.7 to 104.3	132.8 to 147.0
Mean error	-18.3	-5.8	-8.3
Mean percentage error	-4.2%	-6.2%	-6.3%
Total cost			
Observed	$27,362	$8,477	$10,118
Forecast point estimate	$28,698	$9,062	$11,074
95% prediction interval	$26,697 to $30,700	$8,256 to $9,867	$8,081 to $9,692
Mean error	-$1,336	-$585	-$957
Mean percentage error	-4.9%	-6.9%	-9.5%
**2020 Q2**			
Number of services			
Observed	403.7	82.4	101.3
Forecast point estimate	435.6	98.1	144.2
95% prediction interval	418.7 to 452.4	93.3 to 103.0	134.2 to 154.3
Mean error	-31.8	-15.7	-43.0
Mean percentage error	-7.9	-19.1	-42.4
Total cost			
Observed	$26,132	$7,602	$8,242
Forecast point estimate	$27,702	$8,887	$11,008
95% prediction interval	$25,700 to $29,703	$8,081 to $9,692	$10,225 to $11,791
Mean error	-$1,569	-$1,284	-$2,766
Mean percentage error	-6.0%	-16.9%	-33.6%

Supplementary Figure S8: Heatmap of the estimated mean percentage error in number of services provided per provider by each manual therapy profession during the first half of 2020 across Australian States and Territories

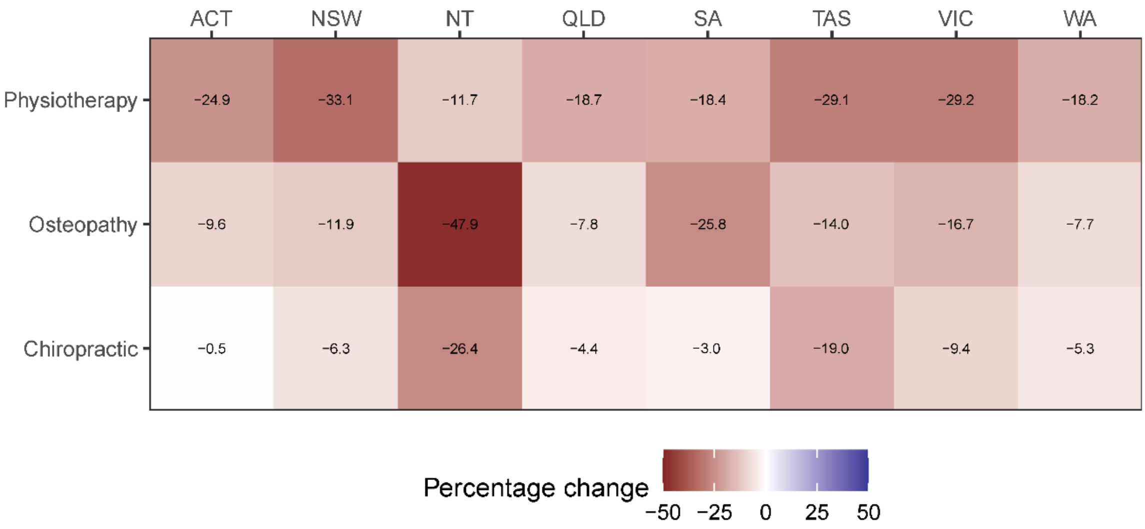

Supplementary Figure S9: Heatmap of the estimated mean percentage error in total cost of services provided per provider by each manual therapy profession during the first half of 2020 across Australian States and Territories

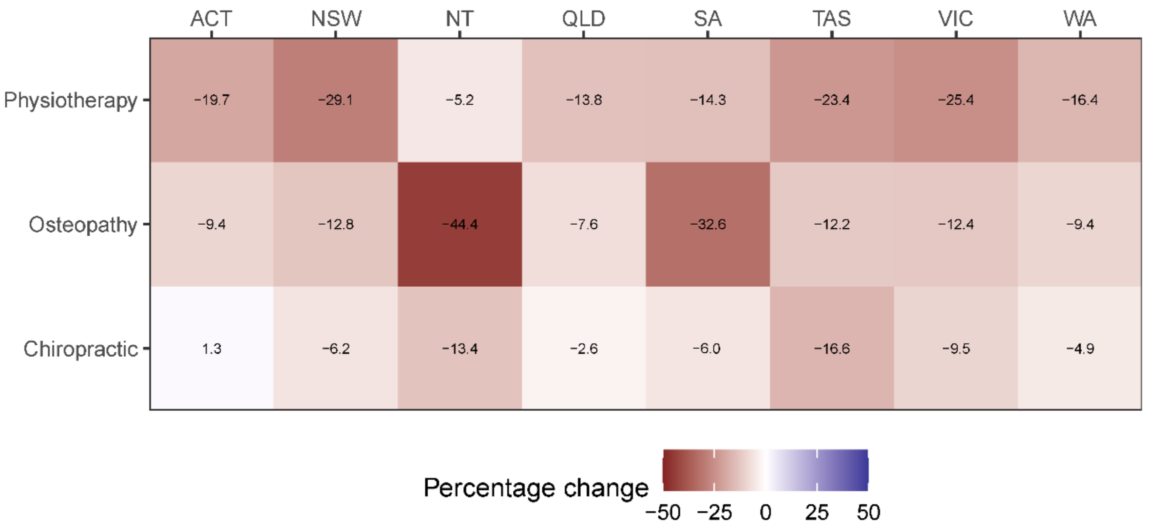

Supplement: Supplementary file 1 [file healthcare-08-00558-s001.pdf]
